# Defective PTEN-induced kinase 1/Parkin mediated mitophagy and neurodegenerative diseases

**DOI:** 10.3389/fncel.2022.1031153

**Published:** 2022-10-20

**Authors:** Megan M. Braun, Luigi Puglielli

**Affiliations:** ^1^Department of Medicine, University of Wisconsin-Madison, Madison, WI, United States; ^2^Waisman Center, University of Wisconsin-Madison, Madison, WI, United States; ^3^Neuroscience Training Program, University of Wisconsin-Madison, Madison, WI, United States; ^4^Department of Neuroscience, University of Wisconsin-Madison, Madison, WI, United States; ^5^Geriatric Research Education Clinical Center, Veterans Affairs Medical Center, Madison, WI, United States

**Keywords:** mitophagy, mitochondria, neurodegeneration, Parkinson’s disease, Alzheimer’s disease, Huntington’s disease, amyotrophic lateral sclerosis, mitochondrial dysfunction

## Abstract

The selective degradation of mitochondria through mitophagy is a crucial process for maintaining mitochondrial function and cellular health. Mitophagy is a specialized form of selective autophagy that uses unique machinery to recognize and target damaged mitochondria for mitophagosome- and lysosome-dependent degradation. This process is particularly important in cells with high metabolic activity like neurons, and the accumulation of defective mitochondria is a common feature among neurodegenerative disorders. Here, we describe essential steps involved in the induction and progression of mitophagy, and then highlight the various mechanisms that specifically contribute to defective mitophagy in highly prevalent neurodegenerative diseases such as Parkinson’s disease, Alzheimer’s disease, Huntington’s disease, and Amyotrophic Lateral Sclerosis.

## Introduction

Mitochondria are highly energetic organelles that play fundamental regulatory roles in multiple cellular events, from bioenergetics to oxidative stress, Ca^+2^ signaling/homeostasis and metabolism. To manage these complex functions, mitochondria undergo many dynamic changes; they can associate with cytoskeleton and move to deliver energy, in the form of ATP, or metabolites where they are needed; they can also undergo cycles of fission and fusion to manage metabolic challenges associated with specific environmental conditions (McBride et al., 2006).

Maintaining mitochondrial functioning is crucial for cellular health, and mitophagy, the selective removal of damaged mitochondria, is emerging as a fundamental cellular strategy to eliminate dysfunctional mitochondria and allow the cell to replenish the pool of healthy mitochondria *via* mitochondrial biogenesis (Roca-Portoles and Tait, 2021). This process is of particular importance in neurons because neurons have high metabolic demands and, as post-mitotic cells, cannot “dilute” damaged mitochondria through cell division (Evans and Holzbaur, 2020b). Importantly, mitochondrial dysfunction and mitophagy defects have been linked to many neurodegenerative disorders (Cai and Jeong, 2020; Cen et al., 2021; Li et al., 2021; Mani et al., 2021; Wang X.-L. et al., 2021).

## Functional overview of PTEN-induced kinase 1/Parkin dependent mitophagy

**FIGURE 1:**
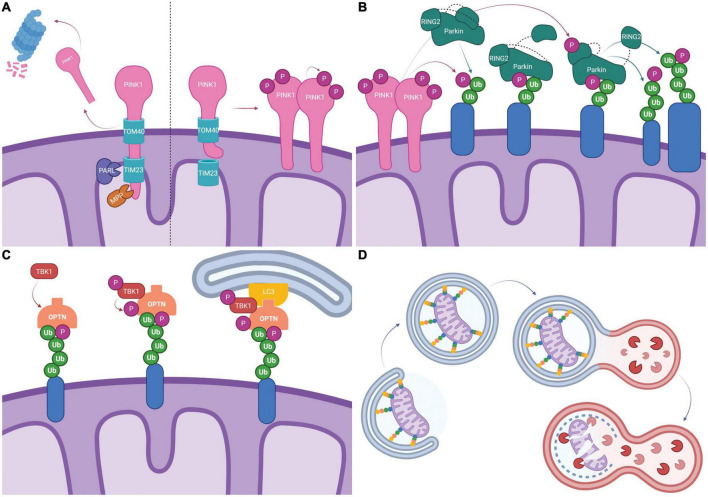
Schematic representation of PINK1/Parkin dependent mitophagy. **(A)** Typically, PINK1 is imported into the mitochondria by TOM40/TIM23 where it is cleaved by mitochondrial proteases PARL and MPP. The c-terminal PINK1 fragment dissociates and is degraded by the proteasome (Left). Following mitochondrial depolarization, PINK1 import *via* TIM23 is blocked by electrostatic repulsion. PINK1 disassociates from TOM40, forms dimers and is activated by auto-phosphorylation (Right). **(B)** Activated PINK1 phosphorylates ubiquitinated mitochondrial proteins (*shown in blue*). Parkin binds phosphorylated ubiquitin which causes a conformational change and exposes the UBL allowing it to be phosphorylated by PINK1. This leads to additional conformational changes and exposes the catalytic site on RING2, fully activating Parkin. Parkin then ubiquitinates mitochondrial proteins, creating more substrates for PINK1 phosphorylation and feed-forward signal amplification. **(C)** Mitophagy receptors, here represented by OPTN, bind ubiquitinated mitochondrial proteins *(shown in blue)*. TBK1 mediated phosphorylation of mitophagy receptors enhances ubiquitin binding. LIR domains on mitophagy receptors recruit the phagophore *via* LC3 binding. **(D)** Elongation of the phagophore leads to the engulfment of mitochondria into mitophagosomes. Mitophagosomes fuse with lysosomes and lysosomal proteases degrade the mitochondria. Created with BioRender.com.

PTEN-induced kinase 1 (PINK1) is a mitochondria-localized serine/threonine kinase that is present at low levels under basal conditions but is stabilized and accumulates on the outer mitochondrial membrane (OMM) as a result of mitochondrial damage and/or depolarization (Matsuda et al., 2010; Narendra et al., 2010; Rakovic et al., 2010; Vives-Bauza et al., 2010). At steady state (normal conditions), the mitochondrial targeting signal (MTS) directs PINK1 to the mitochondria where it transverses the OMM, *via* Translocase of the OMM 40 (TOM40), and the inner mitochondrial membrane (IMM), *via* Translocase of IMM 23 (TIM23) (Wang N. et al., 2020). The positive charge on the MTS allows it to translocate into the matrix where it is cleaved by Mitochondrial Processing Peptidase (MPP) (Wang N. et al., 2020). Subsequently, PINK1 is cleaved by Presenilins-Associated Rhomboid-Like protein (PARL) in the IMM causing PINK1 release into the cytoplasm where it is degraded by the ubiquitin proteasome system (Figure 1A; Wang N. et al., 2020). MPP and PARL play crucial roles in maintaining homeostatic PINK1 localization as inhibition of either MPP or PARL results in abnormally high levels of mitochondrial PINK1 that are sufficient to stimulate the induction of mitophagy (Shi et al., 2011; Greene et al., 2012; Meissner et al., 2015). When mitochondria are damaged and/or depolarized, failure to fully import PINK1 into the matrix disrupts homeostatic PINK1 processing with consequent accumulation and dimerization on the mitochondria surface (Figure 1A; Sekine et al., 2019; Wang N. et al., 2020). The dimerization causes PINK1 to undergo autophosphorylation at Ser228 and Ser402; phosphorylated PINK1 can then phosphorylate ubiquitin and OMM-associated proteins, including Miro, TRAP1 and MFN2, as well as engage Parkin (Figure 1A; Trempe and Fon, 2013; Aerts et al., 2015; Tanaka, 2020; Wang L. et al., 2020). The accumulation of PINK1 on the mitochondria surface stimulates the induction of mitophagy while proteolytic processing and release of PINK1 from the mitochondria surface has the opposite effect (Shi et al., 2011; Greene et al., 2012; Meissner et al., 2015).

The E3 ubiquitin ligase Parkin resides in the cytosol in a closed and inactive conformation under basal conditions; it is activated and recruited to the mitochondria in response to depolarization-induced accumulation of PINK1 on the OMM and its subsequent kinase activity (Geisler et al., 2010a; Matsuda et al., 2010; Rakovic et al., 2010; Vives-Bauza et al., 2010; Tang et al., 2017). Phosphorylated ubiquitin chains on OMM proteins resulting from PINK1 activity serve as receptors for Parkin recruitment to the mitochondria (Shiba-Fukushima et al., 2014; Okatsu et al., 2015). Binding to phosphorylated ubiquitin induces Parkin conformational change to the “open” intermediate state where the interaction between the ubiquitin-like (UBL) and the RING1 domains is disrupted (Ham et al., 2016; Tanaka, 2020; Wang N. et al., 2020). Under this conformation, the UBL is separated from the core and is readily accessible for PINK1-mediated phosphorylation at Ser65 resulting in the fully activated form of Parkin (Shiba-Fukushima et al., 2014; Tang et al., 2017; Tanaka, 2020; Wang N. et al., 2020). Activated Parkin has been shown to catalyze the formation of ubiquitin chains linked by Lys6, Lys11, Lys27, Lys48, and Lys63 (Figure 1B; Geisler et al., 2010a; Bader and Winklhofer, 2020; Tanaka, 2020). This creates a positive feedback loop that amplifies PINK1/Parkin signaling as Parkin-driven ubiquitination of OMM proteins creates more sites for PINK1 phosphorylation, which can then recruit more Parkin. MFN1/2, VDAC1/2/3, Miro, HK1/2, TOMM20, TOMM70A, RHOT1/2, FAF2, and CISD1/2 have all been shown to be ubiquitinated by Parkin (Springer and Macleod, 2016; McLelland et al., 2018; Bader and Winklhofer, 2020).

With ubiquitin binding domains and the ability to recruit the cytosolic components of the autophagy machinery, mitophagy receptors ensure recognition and degradation of damaged mitochondria. Autophagy protein 32 (ATG32) is the only known mitophagy receptor in yeast whereas mammals have several mitophagy receptors including NDP52, OPTN, TAX1BP1, NBR1, and -perhaps- p62 (Kanki et al., 2009; Wong and Holzbaur, 2014a; Evans and Holzbaur, 2020b; Montava-Garriga and Ganley, 2020). The ubiquitin-binding capability of NDP52, OPTN, TAX1BP1, and p62 receptors increases in response to TBK1 phosphorylation, and inhibition of TBK1 activity reduces autophagosome formation (Moore and Holzbaur, 2016; Bader and Winklhofer, 2020; Evans and Holzbaur, 2020b; Montava-Garriga and Ganley, 2020; Wang L. et al., 2020). Mitophagy receptors have confirmed LC3-interacting regions (LIR) suggesting that they can independently recruit LC3β (Figure 1C). However, recent work has highlighted the role of ULK1 complex recruitment as mitophagy is still active in cellular systems where the LC3/ATG8 conjugating system is inactivated (Montava-Garriga and Ganley, 2020; Wang L. et al., 2020). NDP52 directly binds FIP200, a member of the ULK1 complex, but it is currently unknown whether OPTN or TAX1BP1 interact with the ULK1 complex (Montava-Garriga and Ganley, 2020; Wang L. et al., 2020). The different mitophagy receptors have redundant functionality as OPTN, NDP52, and TAX1BP1 all localize to the mitochondria after depolarization or localized ROS generation on the same timescale (Moore and Holzbaur, 2016). However, despite the apparent functional overlap, unique consequences are associated with differential expression of the receptors. The expression of OPTN seems to be of particular importance. Indeed, overexpression of OPTN or NDP52 on a knock-out background of five mitophagy receptors produces the greatest rescue, while deletion of OPTN reduces the speed of mitochondrial engulfment by the autophagosome to a greater extent than NDP52 deletion (Moore and Holzbaur, 2016; Evans and Holzbaur, 2020b). Engagement of LC3β and consequent activation of the cytosolic autophagy machinery, leads to the engulfment of mitochondria into mitophagosomes. Mitophagosomes then fuse with lysosomes and lysosomal proteases degrade the mitochondria (Figure 1D). Additional mechanistic information on the activation and regulation of mitophagy can be found elsewhere (Jishi and Qi, 2022; Li et al., 2022).

## Mitophagy and neurodegenerative diseases

The identification of disease-causing mutations in multiple genes associated with the induction and progression of mitophagy underscores the biological importance of ensuring efficient clearance of dysfunctional (and perhaps even excessive) mitochondria within the cell. Interestingly enough, the great majority of these mutations appear to affect the nervous system. For example, mutations in PINK1 and Parkin are associated with early-onset forms of Parkinson’s Disease (PD) (Borsche et al., 2021; Vizziello et al., 2021) while mutations in MFN2 are associated with axonal forms of Charcot-Marie-Tooth disease 2 (CMT2) and hereditary motor and sensory neuropathies (HMSN) (Zaman and Shutt, 2022). Finally, mutations in OPTN have been linked to primary forms of glaucoma (Sears et al., 2019) as well as amyotrophic lateral sclerosis (ALS) (Benson et al., 2021). In this section, we will describe evidence that supports causative association between different neurodegenerative diseases and dysfunctional mitophagy. We recognize that in some cases, such as with PD-associated mutations in PINK1 and Parkin, the association is direct, while in others, such as with Alzheimer’s disease (AD), the association is only indirect. However, whether defective mitophagy is the cause or the consequence of the disease, it is still important to evaluate its immediate pathologic role and dissect possible disease-mitigating approaches.

### Mitophagy dysfunction in Parkinson’s disease

PD is a neurodegenerative disease clinically characterized by bradykinesia with rest tremor and/or rigidity (Jankovic and Tan, 2020; Bloem et al., 2021). Pathologically, the brains of PD patients display protein aggregates, referred to as Lewy bodies and typically enriched in α-synuclein, and degeneration of nigrostriatal dopaminergic cells (Blauwendraat et al., 2020; Bloem et al., 2021). The resulting imbalance between excitatory and inhibitory input into the basal ganglia causes bradykinesia whereas degeneration of non-dopaminergic pathways is associated with non-motor PD symptoms (Jankovic and Tan, 2020; Bloem et al., 2021). The incidence of PD increases with age. More than 10 million individuals are currently affected by PD worldwide; however, this number is expected to rise significantly due to changes in population age distribution as well as improved diagnostic tools (Blauwendraat et al., 2020; Jankovic and Tan, 2020; Bloem et al., 2021).

Monogenic PD accounts for roughly 3–5% of all cases, and several genes have been identified with high confidence to cause PD (Blauwendraat et al., 2020; Bloem et al., 2021). Though single gene mutations are rare, mitochondrial morphological and functional phenotypes found in patient tissue and in patient derived cell lines are generally conserved in patients with familial and sporadic PD. Decreased fusion and increased fission protein levels, as well as imaging studies quantifying mitochondrial length and branching, support increased mitochondrial fragmentation in PD (Zilocchi et al., 2018; Walter et al., 2019; Yakhine-Diop et al., 2019; Hanss et al., 2021). These same studies also reveal more spherical, swollen mitochondria with less cristae suggesting accumulation of damaged mitochondria (Zilocchi et al., 2018; Walter et al., 2019). Functional studies support these findings as mitochondria in patient-derived cell lines have reduced mitochondrial membrane potential (MMP), ATP production, and respiration as well as increased ROS (Grünewald et al., 2010; Hsieh et al., 2016; Delgado-Camprubi et al., 2017; Walter et al., 2019; Yakhine-Diop et al., 2019, 2021; Wauters et al., 2020; Hanss et al., 2021). Mitochondrial morphological and functional phenotypes have been demonstrated in a wide range of PD-like animal and cell models, including increased fragmentation, disrupted cristae structure, depolarized membrane potential, reduced ATP production, decreased respiration, and increased generation of ROS (Lesage et al., 2016; Villeneuve et al., 2016; Zhang et al., 2016; Chen et al., 2018; Martinez et al., 2018; Chiu et al., 2019; Li et al., 2019; Anand et al., 2020; Liu et al., 2020; Noda et al., 2020; Erustes et al., 2021). A summary of mitochondria alterations associated with PD are listed in Table 1.

**TABLE 1:** Summary of mitochondrial phenotype and mitophagy genes associated with neurodegenerative diseases.

Mitochondria alteration	PD	AD	HD	ALS
Number	X	X	X	
Morphology	X	X	X	X
Fission/fusion	X	X		X
Fragmentation	X	X	X	X
Bioenergetics	X	X	X	X
Motility	X			X
Autosomal recessive mutation	X			
Autosomal dominant mutation	X			
GWAS	X	X		X

Alterations to mitochondrial phenotype that suggest mitochondrial dysfunction is a common feature among PD, AD, HD, and ALS, though the certain aspects of mitochondrial phenotype vary by disease. Disease-associated mutations in core mitophagy machinery, however, are not as universal with mutations only being associated with certain neurodegenerative diseases.

Monogenic, autosomal recessive PD is strongly associated with defective mitophagy as the two most commonly mutated genes are *PARK2*, the gene encoding Parkin, and *PINK1* (in order of prevalence) (Jankovic and Tan, 2020; Wang N. et al., 2020). PD-associated mutations in *PINK1* and *PARK2* have all been shown to inhibit mitophagy, although individual mutations appear to disrupt different steps of the process (Geisler et al., 2010b,a; Lee et al., 2010; Narendra et al., 2010; Rose et al., 2011). PINK1 mutations C125G, I368N, and Q456X impact the ability of PINK1 to accumulate on the mitochondria in response to depolarization, while mutations A168P, H271Q, G309D, and W473X affect Parkin mitochondrial localization without interfering with PINK1 depolarization-induced accumulation (Geisler et al., 2010b; Narendra et al., 2010; Ando et al., 2017; Puschmann et al., 2017; Sekine et al., 2019). PD-associated Parkin mutations can prevent Parkin mitochondrial localization. However, C212Y, C289G, C418R, and C441R mutants tend to aggregate while K27N, R33Q, I44A, R46P, A46P, K211N, T240R, C253Y, Q331X, and G430D mutants remain soluble but are still unable to localize on the mitochondria (Geisler et al., 2010a; Narendra et al., 2010; Rose et al., 2011; Ham et al., 2016). Parkin mutations K161N, A240R, R275W, and T415N have normal mitochondrial recruitment but are deficient in mitochondrial ubiquitination (Geisler et al., 2010a; Lee et al., 2010; Narendra et al., 2010). Interestingly, mutations with impaired Parkin recruitment tend to have a less severe mitophagy defect than mutations that completely inhibit Parkin translocation (Narendra et al., 2010). Although most PINK1 mutations cause PD through recessive inheritance, the inheritance of G411S appears to be dominant. When found in heterodimers, subtle structural changes at the dimer interface of G411S mutated PINK1 propagate structural change to the WT PINK1 and interfere with its ubiquitin kinase activity (Puschmann et al., 2017).

Although less studied, additional genes have been linked to recessive PD with high confidence. They include *DJ-1*, *Fbxo7*, *PLA2G6*, *ATP13A2*, and *VPS13C*. Mutations in each of these genes have been associated with altered mitophagy and defective mitochondrial degradation. Loss of function mutations in *DJ-1* are the third most common cause of recessive PD (Trempe and Fon, 2013). Analysis of rodents lacking DJ-1 revealed reduced AKT signaling and mislocalization of hexokinase 1 from mitochondria to the cytosol (Hauser et al., 2017). Both events appear to block mitochondrial localization of Parkin as well as subsequent ubiquitin phosphorylation (Hauser et al., 2017). *Fbxo7* PD-associated mutations have been linked to reduced Parkin translocation while *PLA2G6* PD-associated mutations have been associated with reduced protein levels of Parkin and BNIP3 (Burchell et al., 2013; Chiu et al., 2019). Deletion of the *C. elegans* ortholog of *ATP13A2* increases lysosomal pH and effectively reduces lysosomal degradative ability because lysosomal proteases require an acidic environment for maturation (Anand et al., 2020). Unlike the other PD associated mutations, modeling of PD-associated VPS13C loss of function appears to cause PINK1 and Parkin accumulation perhaps reflecting a functional downstream block of mitophagy (Lesage et al., 2016). Proteins in the VPS family are involved in vesicular transport and VSP13C is specifically implicated in protein delivery to the lysosome (Lesage et al., 2016). Since VPS13C loss of function mutations are associated with PD, reduced lysosomal protein delivery could potentially alter lysosomal degradative ability and thus mitophagy (Blauwendraat et al., 2020).

*SNCA*, *GBA*, *VPS35*, and *LRRK2* mutations as well as *SNCA* gene duplication events are associated with autosomal dominant forms of PD and appear linked to reduced mitophagy, albeit with conflicting results. This has been clearly shown with *SNCA*, the gene that encodes α-synuclein, where increased PINK1/Parkin activity, reduced Parkin translocation, defective complex I activity, or defective targeting of mitochondria for autophagy have been implicated with the SNCA-PD association (Chinta et al., 2010; Chen et al., 2018; Shaltouki et al., 2018; Erustes et al., 2021). The pathogenic role of *GBA* and *VPS35* mutations appears to be more straightforward with mitochondrial priming defects caused by reduced mitochondrial localization of Parkin, NBR1, and BNIP3L in the case of *GBA* mutations, and defective lysosomal activity in the case of *VPS35* mutations (Li et al., 2019; Hanss et al., 2021). The case for *LRRK2*-associated mutations is quite complex. Some studies suggest that LRRK2 mutations cause increased mitophagy as evidenced by increased mitophagosome formation, increased colocalization of mitochondria and lysosomes, and reduced mitochondrial levels (Yakhine-Diop et al., 2019, 2021). However, a larger body of evidence suggests that mitophagy is actually decreased in LRRK2 mutants. This evidence includes impairment of specific mitophagy steps and direct observation of mitochondrial degradation by utilizing live imaging techniques (Hsieh et al., 2016; Bonello et al., 2019; Korecka et al., 2019; Liu et al., 2020; Wauters et al., 2020; Singh et al., 2021). An inverse relationship between LRRK2 kinase activity and mitophagy has been suggested. Indeed, PD-associated LRRK2 mutations cause increased kinase activity and are associated with decreased mitophagy while genetic or pharmacological inhibition of LRRK2 kinase activity restores mitophagy (Bonello et al., 2019; Korecka et al., 2019; Wauters et al., 2020). However, there is no consensus on how LRRK2 activity mechanistically regulates mitophagy. Studies showing reduced mitophagy and normal autophagy suggest that the defect is in an early mitophagy-specific step (Wauters et al., 2020; Singh et al., 2021). LRRK2 mutations have been proposed to hinder both Parkin mitochondrial recruitment and Miro removal (Hsieh et al., 2016; Bonello et al., 2019). These two mechanistic features are likely linked as Miro is a target for Parkin mediated ubiquitination, and ubiquitinated Miro is degraded by the proteasome (Birsa et al., 2014; Springer and Macleod, 2016). Miro removal halts mitochondrial motility and is thought to be a required step for mitophagy; both Miro removal and mitophagy are delayed in cells with LRRK2 mutations (Birsa et al., 2014; Hsieh et al., 2016). The same delay in Miro removal and mitophagy was also observed in patient-derived fibroblast lines with PINK1 and Parkin mutations as well as lines derived from sporadic PD patients, thus suggesting that delayed Miro removal could be a common mechanism for multiple causes of PD (Liu et al., 2012; Hsieh et al., 2016). Decreased OPTN mitochondrial recruitment is also observed with LRRK2 mutations and –presumably– results from LRRK2-mediated RAB10 phosphorylation. RAB10 is normally found on depolarized mitochondria in close contact with OPTN, and this colocalization is decreased in LRRK2 mutant cells since LRRK2 activity increases levels of phosphorylated RAB10 (Wauters et al., 2020). Alternatively, some studies show an upregulation of initial mitophagy steps such as mitochondrial ubiquitination, p62 recruitment, and mitophagosome accumulation but have an overall decrease in mitochondrial degradation (Liu et al., 2020). This indicates a defect in mitophagy at a converging point with the general autophagy as mitophagosomes and autophagosomes are both degraded the same way. Evidence of reduced autophagic flux includes reduced formation of mature autophagosomes, and reduced lysosomal number, total area, and mean size (Korecka et al., 2019; Walter et al., 2019).

**FIGURE 2:**
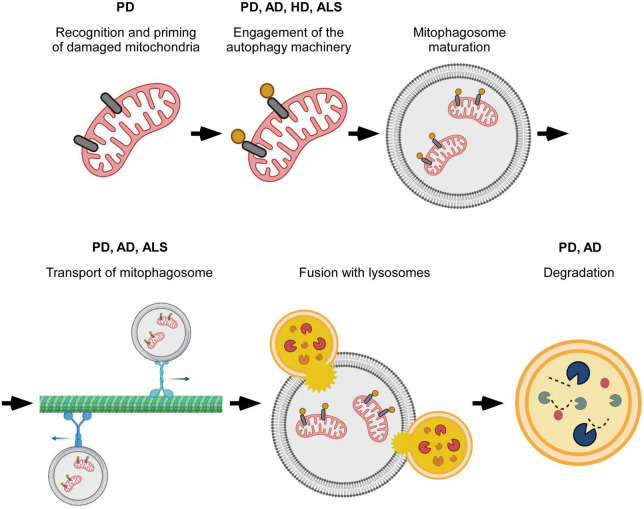
Overview of specific steps in mitophagy that are perturbed in neurodegenerative disease. Mitophagy progression is altered at different stages of the process. Disruptions occur during the initial recognition of damaged mitochondria, formation and transport of mitophagosomes, and degradation of mitochondria by lysosomes. The particular point(s) of mitophagy defect vary with neurodegenerative disease, and, possibly with, disease subtype. Created with BioRender.com.

Mitophagy is also impaired in cell lines derived from sporadic PD patients (Hsieh et al., 2016; Yakhine-Diop et al., 2019). Interestingly, like patients with LRRK2 mutations, sporadic PD patients also have increased LRRK2 kinase activity (Esteves et al., 2015). This would then suggest that shared mechanisms could alter mitophagy in both populations. Indeed, both have impaired Miro removal, and as a result, mitochondrial arrest and mitophagy are delayed (Hsieh et al., 2016). Direct assessment of over 70 patient-derived lines, 43 of which were from patients with sporadic PD, found defective Miro removal in 93% of all the lines (Hsieh et al., 2019). Large scale genetic studies have also helped connect sporadic PD to known mechanisms involved in monogenic PD (Ivatt et al., 2014; Billingsley et al., 2019). For example, the identification of SREBF-1, a previously known risk locus for sporadic PD, in a screen for genes that regulate PINK1/Parkin mediated mitophagy highlights mitophagy dysfunction as a shared mechanistic link between autosomal recessive PD and at least some cases of sporadic PD (Ivatt et al., 2014). Additionally, a GWAS study of sporadic PD patients identified 14 new genes causally associated with PD risk that were involved with various aspects of mitochondrial function including mitophagy, mitochondrial bioenergetics, and mitochondrial proteostasis (Billingsley et al., 2019). The data reported above provides strong support to the conclusion that mitochondrial dysfunction is a key aspect of PD pathophysiology and that it is implicated with both monogenic and sporadic forms of the disease. A schematic summary of mitophagy specific steps that are altered in PD are listed in Figure 2.

### Mitophagy dysfunction in Alzheimer’s disease

AD is the most prevalent neurodegenerative disorder with over 55 million people living with this disease worldwide. Based on the average lifespan, it is estimated that almost 150 million individuals will develop AD by 2050 (Chowdhary et al., 2022). AD is also the sixth most common cause of death accounting for about 30 deaths per 100,000 in 2018 and 2019 (Kochanek et al., 2019; Rajan et al., 2021). The entorhinal cortex and hippocampus are among the first brain regions impacted by AD-associated degeneration, but eventually the disease becomes so widespread to affect both cortical and sub-cortical areas and cause a marked reduction in brain volume (Johnson et al., 2012). Symptoms initially include memory loss and language problems, but eventually basic bodily functions like walking and swallowing are impacted by the progressive neurodegeneration (Alzheimer’s Association, 2021). Accumulation of amyloid-beta (Aβ) into extracellular amyloid (senile) plaques and hyperphosphorylated-Tau into intraneuronal neurofibrillary tangles (NFTs) help distinguish AD from other types of dementias that present with similar symptoms.

A large body of literature suggests that mitochondrial dysfunction is associated with the progression of AD. Mitochondria in AD patient brains have abnormal morphology, including reduced size, swollen shape and abnormal or reduced levels of cristae (Ye et al., 2015; Fang et al., 2019). Higher numbers of mitochondria have also been observed; however, this was only significant in AD brains with high NFT load suggesting that accumulation of mitochondria may occur later in disease progression (Hu et al., 2016). A summary of mitochondria alterations associated with AD are listed in Table 1. The above changes in mitochondrial morphology are consistent with altered mitophagy flux and could either reflect increased mitophagy as damaged parts or damaged organelles need to separate from the larger mitochondrial network for mitophagy to occur, or could reflect decreased mitophagy as the accumulation of damaged mitochondria may be evidence of a degradation blockage. The observed structural changes are also associated with functional changes as hippocampal tissue from AD patients displays signs of energy deficit (Fang et al., 2019). Gene expression studies revealed reduced expression of autophagy- and mitophagy-associated genes in AD patient brains at the mRNA (*ATG12*, *ATG5*, *BECN1*, *OPTN*, *ULK1*, *AMBRA1*, *BNIP3*, *BNIP3L*, *FUNDC1, VDAC1*, *VCP*) and protein (PINK1, BCL2L13, BNIP3L, p-TBK1, p-ULK1) level (Martín-Maestro et al., 2017; Fang et al., 2019). Imaging studies looking at the colocalization of the mitochondrial marker TOMM20 and lysosomal marker LAMP2 indicate reduced mitophagy in AD patient brains (Fang et al., 2019). This finding coexists with an increase in autophagic vesicles, suggesting that the progression of mitophagy, rather than the “marking” of mitochondria for mitophagic degradation, is affected (Ye et al., 2015). Interestingly, PINK1/Parkin tagging of mitochondria for degradation appears to be upregulated in the brain of AD patients as reflected by both higher total protein levels and higher association with mitochondria in late stage AD; this might potentially reflect an attempt to compensate for the dysfunctional or inefficient mitophagy (Ye et al., 2015; Martín-Maestro et al., 2016). Consistently, pSer65-Ub, a specific marker of PINK1-Parkin pathway activity, is increased in AD brains (Narendra, 2021). Similar changes in mitochondrial morphology, ATP levels, and expression of autophagy- and mitophagy-related genes are observed in induced pluripotent stem cells (iPSCs) derived from familial and sporadic AD patients, thus, indicating the importance of mitochondrial dysfunction regardless of disease etiology (Fang et al., 2019).

Models for studying AD that mimic its characteristic accumulation of NFTs and amyloid plaques recapitulate the same dysfunctional mitochondria phenotypes observed in patient-derived samples. Mice with overexpression of human Tau (hTau) and *C. elegans* with phosphomimetic mutations in single-copy expression of hTau isoform 0N4R show similar mitochondrial changes, including increased numbers of mitochondria and more fragmented mitochondria (Hu et al., 2016; Guha et al., 2020). The expression levels of transcription factors that regulate mitochondrial biogenesis, such as PGC-1α, and TFAM, are unchanged in both cellular and mouse models of hTau overexpression, supporting the argument that the observed accumulation of mitochondria results from a failure of mitophagy and not from the upregulation of the essential mitochondrial biogenesis machinery (Hu et al., 2016). Multiple studies have shown that Tau negatively regulates stress-induced mitophagy either by reducing or completely preventing mitophagy, depending on the model and stressor used (Hu et al., 2016; Cummins et al., 2019; Guha et al., 2020). Different models of Tau overexpression have found conflicting results regarding the impact of Tau overexpression on MMP at basal conditions and in response to stress, and have thus suggested different mechanisms for Tau-driven mitophagy interference. Tau overexpressing cells that exhibit reduced depolarization in response to CCCP also have increased Tau accumulation in the OMM, thus suggesting that Tau interferes with MMP through this mislocalization (Hu et al., 2016). The changes in MMP functionally impact mitophagy by decreasing the voltage-dependent mitochondrial localization of PINK1 and Parkin (Hu et al., 2016). Alternatively, Tau might affect mitophagy by binding and “trapping” Parkin in the cytosol (Cummins et al., 2019).

AD models based on APP (amyloid precursor protein) or Aβ overexpression, or Aβ exposure also show increasingly fragmented mitochondrial networks with higher total numbers of mitochondria and reduced mitochondrial length likely caused by a shift in mitochondrial fission/fusion dynamics (Ye et al., 2015; Hu et al., 2016; Martín-Maestro et al., 2017; Castellazzi et al., 2019). These mitochondria are dysfunctional, exhibiting a reduced membrane potential, decreased respiration rates, and increased release of reactive oxygen species (Ye et al., 2015; Han et al., 2017, 2020; Sorrentino et al., 2017). Expression changes at the mRNA and protein levels suggest that decreased mitochondrial biogenesis and reduced mitophagy both play a role in the accumulation of dysfunctional mitochondria (Manczak et al., 2018; Reddy et al., 2018). Reduced retrograde transport of axonal mitophagosomes may contribute to the mitophagy deficit observed in the brains of transgenic mice overexpressing mutant versions of human APP associated with hereditary/familial forms of AD; mitophagosomes accumulate in presynaptic terminals separated from lysosomes that are concentrated in the soma of neurons (Han et al., 2020). Changes in axonal transport dynamics that would support increased overall movement toward the soma, increased retrograde transport and decreased anterograde transport, have also been described. The accompanying accumulation of somal mitophagosomes indicates impaired lysosomal degradation rather than defects in axonal transport (Ye et al., 2015). It is worth noting that the above mentioned studies observed axonal transport on different time scales (100 s vs. 9 min) which could contribute to whether the accumulation of mitophagosomes was respectively, observed in the axon or in the soma (Ye et al., 2015; Han et al., 2020). Expression of DISC1, which was recently discovered to function as a mitophagy receptor, is decreased in post mortem brains of AD patients and in symptomatic APP/PS1 mice indicating a different type of priming failure that is functionally relevant in AD. Indeed, rescuing DISC1 expression reduces Aβ plaque accumulation and synaptic loss, and improves behavioral performance on cognitive tests in APP/PS1 mice (Wang Z.-T. et al., 2019). A schematic summary of mitophagy specific steps that are altered in AD are listed in Figure 2.

### Mitophagy dysfunction in Huntington’s disease

Huntington’s disease (HD) is a rare, monogenic, autosomal dominant neurodegenerative disorder affecting an estimated 5–10 people per 100,000 that is caused by expansion of the polyglutamine (polyQ) region in the huntingtin protein (HTT) (Labbadia and Morimoto, 2013). Trinucleotide repeats of 36 or more are pathogenic with repeat lengths 40 or greater causing full penetrance, and repeat lengths of 36–39 causing incomplete penetrance. People with fewer than 35 repeats usually do not develop HD, although carrying 27–35 repeats may lead to some HD-like symptoms, presumably caused by somatic expansion (Labbadia and Morimoto, 2013; Stoker et al., 2022). Mutant HTT (mHTT) forms aggregates and exhibits pathogenicity through gain-of-function rather than loss-of-function effects (Labbadia and Morimoto, 2013; Stoker et al., 2022). Degeneration is widespread but particularly evident in the GABAergic neurons of the striatum (Stoker et al., 2022). Patients typically experience neuropsychiatric and cognitive symptoms such as apathy, personality changes, and deficits in executive functioning prior to the characteristic choreiform movements which are used to define disease onset (Stoker et al., 2022).

Fibroblasts derived from HD patients exhibit more fragmented mitochondrial morphology and are functionally impaired, with increased ROS generation, decreased MMP, and decreased ATP production (Hwang et al., 2015). Similarly, HD patient iPSC-derived neurons have shorter mitochondria and also exhibit a reduced MMP (Guo et al., 2016). Cellular and animal models of HD recapitulate both the morphological and functional mitochondrial phenotypes observed in the patient derived cell lines in regards to changes in mitochondrial length and fragmentation, ROS and ATP production, and MMP (Hwang et al., 2015; Khalil et al., 2015; Guo et al., 2016; Franco-Iborra et al., 2021). Additional aspects of mitochondrial morphology found altered in HD-like models include functional changes- decreased mitochondrial respiration- and morphological changes- rounder mitochondria with a spheroid shape, abnormal cristae, and an increase in the overall number of mitochondria (Hwang et al., 2015; Khalil et al., 2015; Franco-Iborra et al., 2021). A summary of mitochondria alterations associated with HD are listed in Table 1.

Unchanged mitochondrial protein ubiquitination and decreased LC3 recruitment to the mitochondria suggest that, mechanistically, mitophagy dysfunction occurs downstream of PINK1/Parkin-mediated tagging to damaged mitochondria but upstream of mitophagosome formation (Khalil et al., 2015; Franco-Iborra et al., 2021). Though PINK1 expression is unchanged in HTT mutants, increased expression of PINK1 is capable of rescuing both mitophagy defects and mitochondrial dysfunction (Khalil et al., 2015). PINK1-driven rescue is dependent on Parkin functions, supporting the conclusion that rescue occurs by increasing tagging of damaged mitochondria to compensate for the downstream deficit in mitophagy (Khalil et al., 2015). Targeting a microRNA, miR-302, that is downregulated in cell models of HD also has a rescue effect, improving cell viability and reducing the number and size of mHTT aggregates (Chang et al., 2021). Upregulating miR-302 increases Sirt1 protein levels and AMPK phosphorylation; both events have been shown to increase expression of ATGs (Chang et al., 2021). Thus increasing targeting of damaged mitochondria and increasing the expression of autophagy machinery have both been shown to rescue the mitophagy deficit exhibited by HD models.

Mechanistic studies revealed multiple potential mechanisms for how mHTT can disrupt mitophagy. Specifically, altered engagement of mitophagy receptors (OPTN and NDP52), as well as the autophagy induction machinery (ULK1 and BECN1) have been proposed (Franco-Iborra et al., 2021). HTT normally interacts with OPTN and NDP52, but this interaction is respectively, reduced or abolished between mHTT and OPTN or NDP52 (Franco-Iborra et al., 2021). Consequently, LC3 interaction with the receptors is also reduced indicating improper recognition of mitochondria for degradation (Franco-Iborra et al., 2021). HTT and MTORC1 normally compete for ULK1 binding: ULK1 is inactive when bound to MTORC1 and active when bound to HTT (Franco-Iborra et al., 2021). Cells expressing mHTT exhibit more ULK1 binding with MTORC1 and less ULK1 binding with mHTT, which functionally reduces ULK1 activation as evidenced by the increased levels of phosphorylated ULK1 at Ser757 (Franco-Iborra et al., 2021). HD cell models also show reduced formation of the PtdIns3K complex. This could be due to reduced ULK1 activity as ULK1 phosphorylates and activates the PtdIns3K complex member BECN1. Another alternative or complementary explanation relates to BECN1’s ability to bind expanded polyQ repeats. Normally, BECN1 binding to the polyQ repeat in ATXN3 prevents its degradation. The presence of mHTT could competitively interfere with BECN1-ATXN3 binding and lead to increased degradation of BECN1 (Franco-Iborra et al., 2021). Defects in mitophagy might also occur further downstream. Indeed, increased mitophagosome accumulation along the axon and decreased numbers of acidified autophagosomes closer to the cell body have been described (Wong and Holzbaur, 2014b). Alternatively, there is evidence that excessive mitophagy might result from VCP binding mHTT in HD models (Guo et al., 2016). Normally VCP is necessary for the proteasomal degradation of Mfn1/2 after ubiquitination by Parkin, which blocks mitochondrial fusion as means to alleviate depolarization (Tanaka et al., 2010). However, VCP could also stimulate mitophagy by binding LC3 through proposed LIR domains (Guo et al., 2016). A schematic summary of mitophagy specific steps that are altered in HD are listed in Figure 2.

### Mitophagy dysfunction in amyotrophic lateral sclerosis

ALS is a neurodegenerative disease that impacts both the brain and the spinal cord (Shatunov and Al-Chalabi, 2021; Siddique and Siddique, 2021). There are approximately 1.75–3 new cases per 100,000 persons every year (Masrori and Van Damme, 2020). Patients are typically in their mid-fifties to mid-sixties when early symptoms manifest (Siddique and Siddique, 2021). Most patients initially experience asymmetric and focal weakness in distal limb muscles, more commonly in the dominant hand or tibial muscles (Masrori and Van Damme, 2020; Siddique and Siddique, 2021). The remaining one-third of ALS patients initially present bulbar muscle weakness which results in dysarthria (difficulty speaking) and/or dysphagia (difficulty swallowing) (Masrori and Van Damme, 2020; Siddique and Siddique, 2021). As the disease progresses, muscle weakness and atrophy increasingly spread to adjacent regions and can eventually result in paralysis (Masrori and Van Damme, 2020; Shatunov and Al-Chalabi, 2021; Siddique and Siddique, 2021). Median survival is 3–5 years after symptoms onset, and death is frequently caused by respiratory muscle failure (Masrori and Van Damme, 2020; Siddique and Siddique, 2021).

Though ALS was traditionally characterized as familial or sporadic, convention is switching to classifying ALS as “genetic ALS” or “ALS of unknown cause” because genetic testing has revealed that 14% of patients with no family history have mutations in ALS genes and 20% of patients with family history lack mutations in ALS genes (Shatunov and Al-Chalabi, 2021; Siddique and Siddique, 2021). There are currently 20–30 well-established genes associated with increased ALS risk. The top five, by prevalence, are *C9ORF72*, *SOD1*, *FUS*, *TARDBP*, and *TBK1* (Masrori and Van Damme, 2020; Shatunov and Al-Chalabi, 2021; Siddique and Siddique, 2021). In 95–97% of ALS patients, TDP-43, the protein product of *TARDBP*, is found mislocalized from the nucleus into cytoplasmic aggregates (Masrori and Van Damme, 2020; Shatunov and Al-Chalabi, 2021). TDP-43 pathology has been found in all types of ALS patients excluding patients with *FUS* and *SOD1* mutations where instead, aggregates of the respective mutated proteins accumulate (Masrori and Van Damme, 2020; Shatunov and Al-Chalabi, 2021). Studies utilizing post-mortem brain tissue from patients with TDP-43 proteinopathy and fibroblast lines derived from patients harboring ALS-associated mutations in *TARDBP* or *C9ORF72* demonstrate mitochondrial abnormalities (Onesto et al., 2016; Wang P. et al., 2019). Mitochondria appear rounder, more fragmented, and display swollen or partial to complete loss of cristae (Onesto et al., 2016; Wang P. et al., 2019). Morphological changes in mitochondrial fragmentation, shape, and cristae structure are reproduced in ALS cellular and animal models (Xu et al., 2010; Wang et al., 2013; Wang P. et al., 2019; Evans and Holzbaur, 2020a; Jun et al., 2020). Patient-derived fibroblasts also provide evidence that mitochondrial function is altered in ALS in addition to morphology (Onesto et al., 2016). Some of the observed functional changes appear to be dependent on the specific ALS-associated mutation. Cells harboring *TARDBP* and *C9ORF72* mutations showed significant changes in fission/fusion dynamics, membrane polarization, and mitochondrial mass, but the directionality of the changes was different between the two models (Onesto et al., 2016). Mirroring this, TDP-43 ALS models generally point to increased mitochondrial fission and decreased fusion (Xu et al., 2010; Wang et al., 2013). Though the reverse has also been observed, increased fission and decreased fusion is consistent with the previously mentioned increased mitochondrial fragmentation, strengthening the case for these changes (Wang et al., 2013; Onesto et al., 2016; Davis et al., 2018; Wang P. et al., 2019; Jun et al., 2020). Patients with *C9ORF72* hexanucleotide expansion appear to exhibit a unique functional mitochondrial phenotype: TDP-43, SOD1, and OPTN based ALS models all have depolarized MMP whereas MMP is hyperpolarized in C9ORF72 models (Hong et al., 2012; Wang et al., 2013; Xie et al., 2015; Onesto et al., 2016; Wang P. et al., 2019; Evans and Holzbaur, 2020a; Jun et al., 2020). C9ORF72 models are also unique in having increased ATP content and maximum oxygen consumption rate while other ALS models exhibit decreased ATP synthesis and maximum oxygen consumption (Mattiazzi et al., 2002; Onesto et al., 2016; Wang P. et al., 2019). However, these differences could potentially be explained by increased biogenesis in C9ORF72 models, as evidenced by increased PGC1-α, in attempt to compensate for dysfunctional mitochondria (Onesto et al., 2016). Increased levels of mitochondrial ROS across models suggests a shared failure of mitochondrial function (Mattiazzi et al., 2002; Hong et al., 2012; Wang et al., 2013; Xie et al., 2015; Onesto et al., 2016; Wang P. et al., 2019; Tak et al., 2020). A summary of mitochondria alterations associated with ALS are listed in Table 1.

TDP-43 colocalizes with mitochondrial markers in spinal cord and brain tissue from ALS patients and in patient-derived fibroblast lines containing *TARDBP* mutations, suggesting a potential mechanism by which TDP-43 could affect mitochondrial function (Wang et al., 2016; Wang P. et al., 2019). This finding has been replicated in primary mouse cell lines and standard cell lines that exogenously express human TDP-43 (hTDP-43) harboring ALS-associated mutations as well as C-terminal TDP-43 fragments (Hong et al., 2012; Wang et al., 2013; Wang P. et al., 2019; Jun et al., 2020). Importantly, the C-terminal fragments resulting from proteolytic cleavage of TDP-43 are found in TDP-43 cytoplasmic inclusions, while exogenous expression of hTDP-43 in mice results in ALS-like motor phenotypes including tremors, difficulty walking, and abnormal hindlimb clasping in addition to cytoplasmic TDP-43 aggregates in spinal cord motor neurons (Shan et al., 2010; Xu et al., 2010; Jun et al., 2020). Furthermore, preventing mitochondrial TDP-43 localization through deletion of the M1 region was able to normalize mitochondrial length, membrane potential, oxygen consumption rate, and ATP synthesis in cells overexpressing WT or mutant TDP-43, thus providing evidence that mitochondrial localization of TDP-43 contributes to mitochondrial dysfunction (Wang et al., 2016). Exogenous expression of TDP-43 or C-terminal TDP-43 fragments also appeared to enhance mitophagy as evidenced by increased Parkin recruitment to mitochondria and increased levels of LC3-II (Hong et al., 2012; Davis et al., 2018; Jun et al., 2020). However, it is unclear if this increase results in increased degradation of mitochondria since TDP-43 expression also increases the percentage of stationary mitochondria in axons and neurites (Cai et al., 2012; Wang et al., 2013; Evans and Holzbaur, 2020a).

Mutations to *SOD1* are the second most common cause of genetic ALS and have been the subject of much research as *SOD1* was the first genetic cause of ALS discovered (Rosen et al., 1993; Masrori and Van Damme, 2020; Shatunov and Al-Chalabi, 2021). Mutations to *SOD1* seem to impact mitophagy in addition to mitochondrial morphology and functionality, as discussed earlier, though the specific mechanistic components (and consequences) are not as clear. Evidence supporting increased mitophagy resulting from ALS-associated *SOD1* mutations includes increased p62 recruitment to the mitochondria suggesting increased mitochondrial priming, decreased levels of mitochondrial proteins suggesting lower levels of mitochondria, and, most convincingly, increased mitophagy as demonstrated by the mt-Keima reporter in *SOD1* mutant mice (Palomo et al., 2018). Other studies have found that *SOD1* mutant motor neuron cell bodies and presynaptic terminals at the neuromuscular junctions (NMJs), respectively, have increased mitophagosome formation or no significant changes from WT which appears consistent with increased mitophagy in some regions of the cell (Rogers et al., 2017). However, because the number of damaged mitochondria is greater in *SOD1* mutant presynaptic NMJ terminals than in WT, and the number of damaged mitochondria increases faster than mitophagosomes in *SOD1* mutant motor neuron cell bodies, a mitophagy defect is manifest (Rogers et al., 2017). Additional evidence pointing to a mitophagy defect in *SOD1* mutant models includes reduced levels of mitophagy proteins BNIP3, PINK1, and Parkin, fewer mitophagosomes, higher levels of mitochondria after stimulation with the mitophagy inducer CCCP, accumulation of p62-associated mitochondria, and abnormal degradative vesicles containing mitochondria (Xie et al., 2015; Rogers et al., 2017; Tak et al., 2020). One potential explanation for the observed mutant SOD1-associated mitophagy defects is its ability to interact with mitophagy receptor OPTN leading to the sequestration of OPTN to SOD1 aggregates, which would hinder the recognition of damaged mitochondria (Tak et al., 2020). Mutant SOD1 has also been shown to interact with retrograde trafficking protein dynein and to impair the interaction between dynein and endosomal adaptor trafficking protein Snapin (Xie et al., 2015). As a result, live imaging indicates hindered maturation of endocytic vesicles and autophagosomes, and reduced levels of mature lysosomes as this process requires axonal transport in neurons (Xie et al., 2015). Overexpression of Snapin restores the Snapin dynein interaction and rescues the lysosomal phenotype seen in *SOD1* mutants in addition to restoring mitochondrial morphology and functionality, and ameliorating the ALS-like symptoms observed in the mouse (Xie et al., 2015).

ALS-associated mutations in *TBK1* and the functionally related protein OPTN have clearly established roles in mitophagy with OPTN recognizing damaged mitochondria through its ubiquitin-binding domain, and TBK1 phosphorylating OPTN and increasing the ubiquitin-binding ability of OPTN (Wong and Holzbaur, 2014a; Moore and Holzbaur, 2016; Bader and Winklhofer, 2020; Evans and Holzbaur, 2020b; Montava-Garriga and Ganley, 2020; Wang L. et al., 2020). As such, it is not difficult to imagine how mutations to these proteins have the potential to alter mitophagy. Two ALS-associated mutations in *OPTN* have been found to likely affect its ubiquitin-binding ability either indirectly through changes to the dimeric structure of OPTN or directly through mutations in the ubiquitin-binding domain (Li et al., 2018). The latter has been shown to impact mitophagy resulting in decreased OPTN recruitment to damaged mitochondria, slower mitophagosome formation, and increased mitochondrial levels after mitophagy stimulation (Wong and Holzbaur, 2014a; Moore and Holzbaur, 2016; Evans and Holzbaur, 2020a). Another ALS-associated *OPTN* mutation, which results in a premature stop that eliminates the ubiquitin-binding domain, also has impaired mitophagosome formation indicating the importance of OPTN as a mitophagy receptor (Maruyama et al., 2010; Moore and Holzbaur, 2016). Multiple patient mutations in *TBK1* have reduced or completely abolished OPTN binding activity and expectedly, given the role of TBK1 in enhancing OPTN function, inhibit mitophagosome formation (Freischmidt et al., 2015; Moore and Holzbaur, 2016). A schematic summary of mitophagy specific steps that are altered in ALS are listed in Figure 2.

## Conclusion

This review summarizes the mechanisms of PINK1/Parkin dependent mitophagy and describes how mitophagy is altered in multiple neurodegenerative diseases. Despite strong evidence showing that defective mitophagy is linked to neurodegenerative diseases, there are evident limitations. In patient-based studies, the disease has already been progressing for years before mitophagy can be analyzed, making it impossible to determine causality. Cell and animal model-based studies begin with genetic alterations (i.e., expression of WT or mutant versions of human genes) designed to mimic a specific disease. As such, the observed changes in the induction and/or progression of mitophagy must be viewed as a downstream consequence of the genetic event.

The genetic alterations associated with the individual diseases likely impact many aspects of cell biology beyond mitophagy and thus, rescuing mitophagy is unlikely to rescue the full spectrum of induced changes. However, solely rescuing mitophagy-associated defects may be beneficial as many different treatments that rescue mitophagy have been shown to improve lifespan, disease-associated behavior, and protein aggregate accumulation in various neurodegenerative disease models (Wang et al., 2018; Wu et al., 2018; Fang et al., 2019; Li and Chen, 2019; Moskal et al., 2020; Yang et al., 2020; Zhou et al., 2020; Chen et al., 2021; Tjahjono et al., 2021; Wang H. et al., 2021). Additional information on treatments targeting mitophagy is reviewed in Clark et al. (2021), Masaldan et al. (2022), and Pradeepkiran et al. (2022).

Another source of debate is the directionality of the mitophagy-associated alterations. As discussed above, there are conflicting claims for whether specific molecular steps involved with the induction and progression of mitophagy are downregulated or upregulated in AD, HD, ALS, and selected PD cases. Indeed, evidence of upregulated early steps may actually result from blockage in a downstream process. However, it is also possible that mitophagy is upregulated in some patients and downregulated in others, as excessive and insufficient mitophagy can both negatively affect cell energy metabolism. Excessive mitophagy depletes the mitochondrial pool and thus limits energy production through oxidative phosphorylation whereas insufficient mitophagy fails to clear defective mitochondria which generate high levels of ROS and can trigger apoptosis (Montava-Garriga and Ganley, 2020). Accordingly, mitophagy-based treatments would likely need to be considered on a disease-by-disease or even a patient-by-patient basis. It is also important that treatments do not overcompensate and negatively impact mitochondria homeostasis in the other direction. Given that different steps along the entire process of mitophagy have been proposed to be altered in neurodegenerative disease, the mechanism of action for mitophagy-based treatments will likely determine what population will respond to a given treatment. For example, increasing mitochondrial tagging for degradation may not be beneficial when lysosomal degradative ability is responsible for reduced mitophagy.

One intricately intertwined factor that was not discussed in this review, due to the specific PINK/Parkin focus, is the mitochondrial dysfunction that accompanies aging. Although the complexity of aging can potentially affect different molecular aspects of mitophagy, a common element appears to be a decreased efficiency of oxidative phosphorylation, which generates less ATP and more reactive oxygen species. For further review of the role of aging in mitochondrial dysfunction and mitophagy (see Morton et al., 2021; Song et al., 2021; Tran and Reddy, 2021).

Though additional research is needed, mitophagy dysfunction is a common feature among several neurodegenerative diseases and could potentially be a compelling therapeutic target.

## Author contributions

MB wrote the manuscript. LP revised the manuscript. Both authors contributed to the article and approved the submitted version.

## Abbreviations

Aβ: amyloid-beta
AD: Alzheimer’s disease
ALS: Amyotrophic Lateral Sclerosis
APP: amyloid precursor protein
HD: Huntington’s disease
hTau: human Tau
hTDP-43: human TDP-43
HTT: huntingtin protein
iPSCs: induced pluripotent stem cells
IMM: inner mitochondrial membrane
LIR: LC3-interacting regions
MMP: mitochondrial membrane potential
MPP: mitochondrial processing peptidase
MTS: mitochondrial targeting signal
mHTT: mutant huntingtin protein
NFTs: neurofibrillary tangles
NMJs: neuromuscular junctions
OMM: outer mitochondrial membrane
PARL: presenilin-associated rhomboid-like protein
PD: Parkinson’s disease
PINK1: PTEN-induced kinase 1
polyQ: polyglutamine
TDP-43: TAR DNA-binding protein 43
TIM23: translocase of the inner mitochondrial membrane 23
TOM40: translocase of the outer mitochondrial membrane 40
UBL: ubiquitin-like.
